# Development and validation of a machine learning model for predicting stroke-associated pneumonia in older patients with acute ischemic stroke

**DOI:** 10.3389/fneur.2026.1801193

**Published:** 2026-06-10

**Authors:** Wen-Jie Chu, Si-Ran Zhang, Qi-Lun Lai, Jing-Ying Yu, Yi-Qian Xu

**Affiliations:** 1 Department of Hospital Infections, Zhejiang Hospital, Hangzhou, China; 2 Department of Neurology, Zhejiang Hospital, Hangzhou, China

**Keywords:** acute ischemic stroke, machine learning, prediction model, SHapley Additive exPlanations, stroke-associated pneumonia

## Abstract

**Objective:**

Stroke-associated pneumonia (SAP) is a common and serious complication in older patients with acute ischemic stroke (AIS). However, early identification of high-risk patients remains challenging. This study aimed to develop and validate an interpretable machine learning model for predicting SAP risk in older AIS patients.

**Methods:**

This retrospective study included 1,011 eligible patients (aged ≥65 years) with AIS who were consecutively admitted to Zhejiang Hospital in China from September 1, 2018, to December 31, 2023. A total of 1,011 patients were randomly divided into training and testing sets (7:3 ratio). Demographics, comorbidities, laboratory test results, and admission assessments were collected to evaluate the risk of SAP. The synthetic minority oversampling technique (SMOTE) was used to address the imbalanced training data. The Least Absolute Shrinkage and Selection Operator (LASSO) regression was used to filter the predictive features. Eight machine learning models, including Logistic Regression (LR), Support Vector Machine (SVM), Light Gradient Boosting Machine (LightGBM), eXtreme Gradient Boosting (XGBoost), Categorical Boosting (CatBoost), Gradient Boosting Decision Tree (GBDT), Multi-layer Perceptron (MLP), and Random Forest (RF), were applied to identify the best prediction model. The optimal model was interpreted using the SHapley Additive exPlanations (SHAP).

**Results:**

SAP incidence was 18.79%. LASSO identified 12 predictive features. The SVM demonstrated acceptable and stable predictive performance, achieving an accuracy of 0.773, sensitivity of 0.667, specificity of 0.798, F1 score of 0.524, Brier score of 0.156, and AUC of 0.794 (95% CI: 0.748–0.839) in the test set. SHAP analysis identified key factors influencing model predictions. An online platform was developed for clinical use.

**Conclusion:**

This study demonstrates that an interpretable SVM-based machine learning model can effectively predict the risk of SAP in older patients with AIS using routinely available clinical and laboratory data. SHAP analysis further improved the model’s clinical interpretability by elucidating feature contributions. Our online prediction platform could serve as a promising tool for identifying high-risk older patients and facilitating the early prophylactic management of SAP.

## 1 Introduction

Stroke-associated pneumonia (SAP) specifically refers to the emergence of a lower respiratory tract infection within 7 days of stroke onset, which is one of the most common complications of stroke (1). SAP is associated with adverse outcomes and prolonged hospital stays (2, 3). Compared with younger patients, SAP in older patients leads to a worse prognosis and elevated mortality rates, imposing substantial economic burdens on the global aging society (4). It has been reported that the proportion of China’s population aged ≥65 years will reach 38.1% by 2050 (5, 6).

Though the incidence of stroke has declined in some regions, the absolute number of stroke cases continues to increase due to population aging and demographic growth, further reinforcing the need for predictive models specifically tailored to older patients (7). Consequently, the ability to predict SAP risk in older patients could provide strong support for timely decision-making and intervention.

Although several studies have examined the risk factors related to SAP and hospital outcomes, existing risk assessment tools for SAP remain limited in terms of predictive accuracy and generalizability (8–10). In clinical practice, traditional scoring systems, such as the AIS-APS, PANTHERIS, and A^2^DS^2^ scores, have been developed to predict the risk of SAP (11–13). However, these scales are intended for a single assessment at admission and are mostly insufficient for continuous SAP-risk evaluation and may not fully reflect the changes after stroke (14).

With the development of machine learning (ML) methods and the widespread adoption of electronic health records (EHR), some prediction models have already been used to predict SAP (15–19). Compared with traditional analysis techniques, machine learning methods can be used to analyze high-dimensional data and extract nonlinear and seemingly irrelevant factors that are difficult to identify with conventional analysis methods, such as linear regression, thereby allowing for more accurate feature selection (20). However, previous studies have primarily focused on an unselected general population. Despite the growing burden of stroke in aging populations, early identification of SAP specifically in older AIS patients remains unknown. Most prior studies have been developed in general stroke populations without accounting for the distinct risk profile of older patients (6). This study aimed to develop and validate an explainable machine-learning model for identifying the risk of SAP in older patients with AIS using routinely available clinical features.

## 2 Materials and methods

### 2.1 Study population

This study was conducted at Zhejiang Hospital in Hangzhou, China. The hospital operates a certified stroke unit that provides various medical services, including acute stroke care, intravenous thrombolysis, endovascular therapy, intensive monitoring, and early rehabilitation. This single-center retrospective study included all AIS patients aged ≥65 years who were admitted to Zhejiang Hospital between September 1, 2018, and December 31, 2023. Eligible patients were those ≥65 years old who underwent cranial computed tomography (CT) or brain magnetic resonance imaging (MRI) within the first 24 h of admission.

The improved diagnostic criteria for stroke-associated pneumonia are based on the guidelines from the Centers for Disease Control and Prevention (CDC) (21). According to the guideline, the following patients were excluded: (1) patients with diseases that have similar clinical manifestations of pneumonia, (2) only hospital-acquired pneumonia was considered, and patients with pneumonia before the stroke were excluded, (3) patients <65 years old and with clinical signs of infection at admission, and (4) patients with incomplete medical records.

### 2.2 Data collection and variables

After thoroughly surveying potential risk factors for SAP identified in previous studies, and discussing the information available in the hospital EHR system with physicians, the following variables, collected at admission, were included: (1) demographics, including age, sex, smoking, alcohol and Proton Pump Inhibitor (PPI) use, (2) comorbidities, including hypertension, diabetes, atrial fibrillation, chronic obstructive pulmonary disease (COPD), hyperlipidemia, heart failure, coronary heart disease (CHD), (3) laboratory test results: albumin, neutrophil count, lymphocyte count, C-reactive protein (CRP), red cell distribution width (RDW), red blood cell count (RBC), low-density lipoprotein (LDL), high-density lipoprotein (HDL), alkaline phosphatase (ALP), total bilirubin, indirect bilirubin, total cholesterol, blood urea nitrogen (BUN), neutrophil-to-lymphocyte ratio (NLR), creatinine, uric acid, (4) assessment at admission, including National Institutes of Health Stroke Scale (NIHSS) score, Water Drinking Test (Wada) score (using Kubota water swallow test), venous thromboembolism (VTE) risk score(using Padua Prediction Score to assess VTE risk at admission), and modified Rankin Scale (mRS) score. The NIHSS and mRS scores were treated as continuous variables in all models to enhance efficiency, under the assumption of linearity. This approach is widely used in clinical prediction modeling and has been shown to produce robust results for these scales. Certified doctors determined baseline NIHSS and mRS scores through face-to-face patient interviews. All data were extracted and managed by trained researchers. Patients with any missing values for any of the 32 initial variables were excluded from the study.

### 2.3 Model development and validation

To develop and validate the prediction model, the dataset was randomly partitioned into a training set (70%) and an independent testing set (30%). The training process involved a 5-fold cross-validation to mitigate overfitting. Furthermore, to address the class imbalance between SAP and non-SAP patients, the Synthetic Minority Oversampling Technique (SMOTE) was applied exclusively to the training data (22). *Z*-score normalization was implemented as the first step within a pipeline that also included SMOTE. Both operations were applied exclusively within each cross-validation fold’s training partition; the validation folds and the held-out test set remained in their original distribution without oversampling. Training set performance metrics were reported on the original, non-SMOTE training data to avoid inflated estimates. An additional temporal validation was performed by training on patients admitted from September 1, 2018, to December 31, 2021 (*n* = 707) and testing on those from January 1, 2022, to December 31, 2023 (*n* = 304). The complete preprocessing pipeline, including the exact sequencing of normalization and synthetic minority over-sampling, is presented in Supplementary File 1.

**Figure 1:**
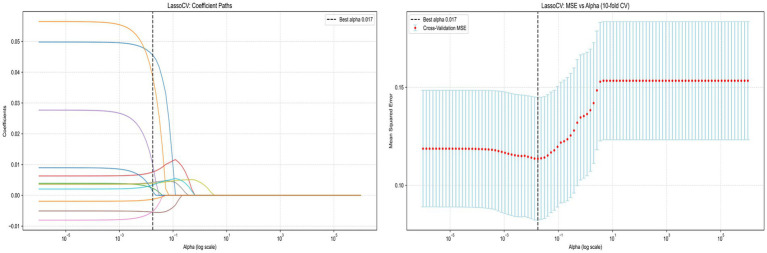
Presentation of the results of the LASSO regression analysis. **(A)** LASSO regression model factor selection: left dashed line represents the optimal lambda value (lambda·min), while the right dashed line marks the lambda value within one standard error of the optimal (lambda.1se); **(B)** LASSO regression model screening variable trajectories.

For feature selection, we also used the least absolute shrinkage and selection operator (LASSO) regression with 5-fold cross-validation to identify characteristics associated with SAP, and significant variables were used as inputs to train and optimize models (Figure 1). Through lasso regression analysis, we finally selected 12 variables, including mRS score, Wada drinking test, VTE score, BUN, NIHSS score, serum albumin, NLR, CRP, age, RDW, total bilirubin, and total cholesterol.

Eight machine learning models were employed based on the selected 12 feature variables. We input these features into 8 different ML algorithms, including LR, SVM, MLP, LightGBM, XGBoost, GBDT, RF, and CatBoost.

A Grid Search was used for hyperparameter optimization that exhaustively explores predefined hyperparameter combinations to identify the optimal configuration for model performance (23). Optimal parameters for each model were extracted through grid search (nfold = 5) to minimize overfitting and enhance model performance. The complete hyperparameter search grid for each model is provided in Supplementary Table 1.

Furthermore, model performance was evaluated using a confusion matrix, F1-score, Brier score, area under the receiver operating characteristic curve (AUC), calibration curves, and decision curve analysis (DCA). The clinical value of the predictive algorithms was assessed based on three criteria: discriminative ability, calibration, and clinical utility. By comparing the algorithms, we selected the best model based on F1-scores and AUC values.

**Figure 2:**
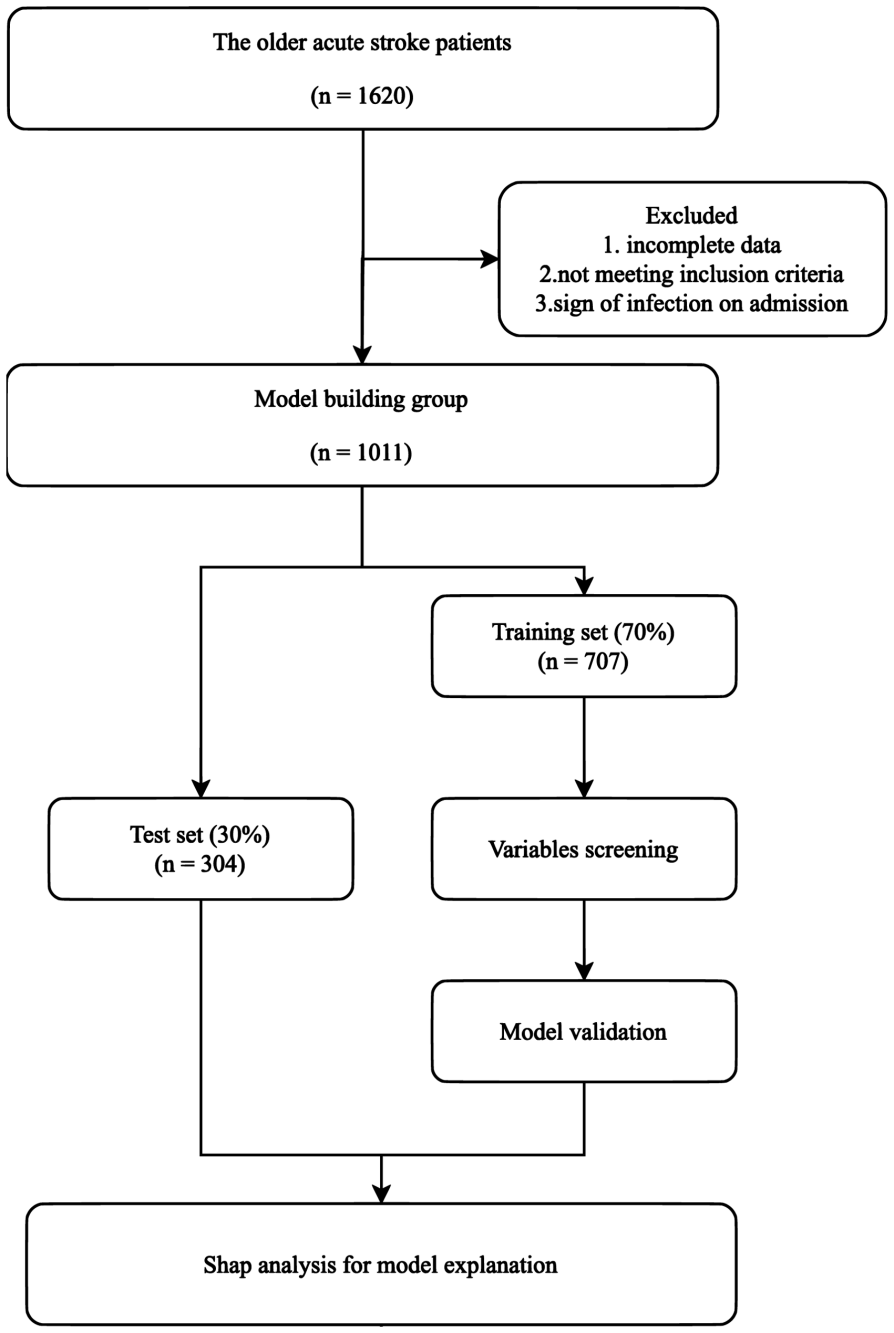
The overall flowchart of the study.

To explain how each feature variable affects and contributes to the model, we employed the SHapley Additive eXplanation (SHAP) method to interpret the best-performing model. The overall study workflow is depicted in Figure 2. Although linear SVM coefficients directly provide global feature importance, SHAP values were computed to provide both instance-level clinical explanations and a framework that ensures consistent feature importance comparison across the eight algorithms.

In order to evaluate the temporal generalizability of the prediction model, an additional temporal validation was performed. Patients admitted between September 2018 and December 2021 (*n* = 707) were used as the training set, and those admitted between January 2022 and December 2023 (*n* = 304) as the test set.

A default probability threshold of 0.50 was used for all binary classification decisions. Sensitivity analyses exploring alternative thresholds, including the threshold optimized by Youden’s index, were conducted and are reported in Supplementary Table 4.

### 2.4 Statistical analysis

All statistical and machine learning analyses were performed using Python version 3.12.4 (Python Software Foundation, Wilmington, Delaware, USA). The study used the D’Agostino-Pearson test to examine the normality of the dataset. Continuous variables are presented as mean ± standard deviation (SD), or median with interquartile range (IQR), and comparisons were made using the student’s *t*-test or the Mann–Whitney *U* test. Categorical variables are expressed as frequencies and percentages, with comparisons conducted using the Chi-square test or Fisher’s exact test, as appropriate. Model performance in terms of AUC was compared pairwise using a bootstrap-based DeLong test (24). The matrix of *p*-values is reported in the Supplementary Table 2. The 95% confidence intervals for AUC and Brier scores were estimated using 2,000 bootstrap replicates. In addition, although mRS and NIHSS are ordinal, they were treated as continuous in the primary analysis, a common practice in stroke predictive modeling. A sensitivity analysis treating them as categorical was performed and yielded similar discriminative performance in Supplementary Table 3. The study is reported in accordance with the TRIPOD Type 2b. A completed TRIPOD+AI checklist is provided in Supplementary File 2.

### 2.5 Ethics statement

Retrospective ethics approval was obtained from the local ethics committee of Zhejiang Hospital in Zhejiang, China (document number: 2024linshen111K) for the study from 2018 to 2023. According to national legislation and institutional requirements, participants do not need to sign an informed consent form. We applied for an informed consent waiver due to the retrospective nature of the study. All patient data were anonymized and maintained with strict confidentiality throughout the research process. All analysis code, preprocessing pipelines, and trained model weights are publicly available at https://github.com/Safnov/1.

## 3 Results

### 3.1 Patient characteristics

Among 1,620 stroke patients, 1,011 were included in this study according to diagnostic criteria for SAP (Supplementary Figure 1). The median age of the 1,011 patients included was 76 (interquartile range, 70–83) years, and 67.6% (*n* = 588) were male. As illustrated in Table 1, significant differences existed in some factors of comorbidities, laboratory tests, and assessment at admission between the training and test sets (*p* < 0.05), whereas no other variables showed significant differences between the demographic characteristics (*p* > 0.05).

**Table 1:** Baseline characteristics of SAP and non-SAP patients with acute ischemic stroke.

Variables	SAP (*n* = 190)	Non-SAP (*n* = 821)	*χ*^2^/*Z* value	*p*-value
Demographics				
Sex, *n* (%)				
Male	103 (54.21)	485 (59.07)	1.5	0.222
Female	87 (45.79)	336 (40.93)		
Age (years) Mean ± SD	80.41 ± 8.09	75.54 ± 7.49	−7.364	0
Smoking, *n* (%)				
Yes	49 (25.79)	243 (29.60)	1.09	0.297
No	141 (74.21)	578 (70.40)		
Drinking, *n* (%)				
Yes	39 (20.53)	205 (24.97)	1.664	0.197
No	151 (79.47)	616 (75.03)		
Comorbidities (%)				
Hypertension, *n*				
Yes	104 (54.74)	441 (53.71)	0.065	0.799
No	86 (45.26)	380 (46.29)		
Diabetes, *n*				
Yes	51 (26.84)	192 (23.39)	1.009	0.315
No	139 (73.16)	629 (76.61)		
PPI, *n*				
Yes	106 (55.79)	513 (62.48)	2.913	0.088
No	84 (44.21)	308 (37.52)		
Atrial fibrillation, *n*				
Yes	44 (23.16)	96 (11.69)	17	0
No	146 (76.84)	725 (88.31)		
*COPD, *n*				
Yes	3 (1.58)	13 (1.58)	N/A	1
No	187 (98.42)	808 (98.42)		
Hyperlipidemia, *n*				
Yes	6 (3.16)	30 (3.65)	0.111	0.739
No	184 (96.84)	791 (96.35)		
Heart failure, *n*				
Yes	10 (5.26)	17 (2.07)	6.05	0.014
No	180 (94.74)	804 (97.93)		
CHD, *n*				
Yes	19 (10.00)	74 (9.01)	0.18	0.676
No	171 (90.00)	747 (90.99)		
Laboratory tests (IQR)				
Albumin, median	37.60 (34.27, 39.79)	39.37 (37.26, 41.40)	−6.339	0
Neutrophil, median	6.10 (4.300, 8.225)	4.10 (3.30, 5.20)	−9.911	0
Lymphocyte, median	1.20 (0.90, 1.60)	1.50 (1.10, 1.80)	−5.307	0
CRP, median	10.05 (2.80, 33.28)	2.06 (0.80, 5.33)	−10.463	0
RDW, median	13.30 (12.80, 14.30)	13.00 (12.60, 13.50)	−5.012	0
RBC, median	4.07 (3.64, 4.47)	4.25 (3.86, 4.61)	−3.718	0
LDL, median	2.22 (1.73, 2.74)	2.45 (1.88, 3.00)	−3.034	0.002
HDL, median	1.06 (0.86, 1.26)	1.06 (0.90, 1.24)	−0.867	0.386
ALP, median	79.00 (65.00, 99.00)	76.00 (64.00, 91.00)	−2.294	0.022
Total bilirubin, median	12.65 (8.77, 18.24)	12.58 (9.42, 16.63)	−0.370	0.711
Indirect bilirubin, median	7.56 (5.26, 11.67)	8.01 (5.97, 11.01)	−0.653	0.514
Total cholesterol, median	3.87 (3.09, 4.44)	4.02 (3.44, 4.72)	−3.180	0.001
BUN, median	6.07 (4.80, 7.80)	5.50 (4.60, 6.70)	−3.387	0.001
NLR, median	4.84 (3.25, 7.67)	2.70 (2.02, 3.91)	−10.054	0
Creatinine, median	73.50 (60.00, 95.00)	74.00 (62.00, 89.00)	−0.332	0.74
Uric acid, median	312.5 (234.0, 377.0)	320.0 (263.0, 395.0)	−2.000	0.046
Assessment at admission (IQR)				
NIHSS score, median	5.00 (2.00, 14.00)	2.00 (1.00, 3.00)	−9.127	0
Wada score, median	1.00 (1.00, 3.00)	1.00 (1.00, 1.00)	−10.191	0
VTE_score, median	2.00 (2.00, 3.00)	2.00 (1.00, 2.00)	−7.493	0
mRS score, median	3.00 (2.00, 4.00)	1.00 (1.00, 3.00)	−11.777	0

SD, standard deviation; IQR, interquartile range; PPI, Proton Pump Inhibitor use; COPD, chronic obstructive pulmonary disease; CHD, coronary heart disease; CRP, C-reactive protein; RDW, red cell distribution width; RBC, red blood cell; LDL, low-density lipoprotein; HDL, high-density lipoprotein; ALP, alkaline phosphatase; BUN, blood urea nitrogen; NLR, neutrophil-to-lymphocyte ratio; NIHSS, National Institute of Health Stroke Scale; Wada, Water Drinking Test (Kubota water swallow test); VTE, venous thromboembolism; mRS, modified Rankin scale.

The incidence of SAP in our cohort was 18.79% (*n* = 190). Differences in variables between patients with and without SAP are presented in Table 1. The table indicated statistically significant differences (*p* < 0.05) between the two patient groups, while no other data showed significant statistical differences (*p* > 0.05).

### 3.2 Model performance and validation

From the point of view of influence factors, the selection of features or variables is crucial in developing prediction models (25). Feature selection was performed using the Least Absolute Shrinkage and Selection Operator (LASSO) regression, which identified 12 predictive factors from the initial 32 variables for model construction. These variables were: mRS score, NIHSS score at admission, VTE risk score, BUN, serum albumin, age, total cholesterol, Wada drinking score, NLR, CRP, RDW, and total bilirubin. Sensitivity analyses treating the NIHSS and mRS as ordinal variables yielded consistent results (Supplementary Table 3), supporting the robustness of the main findings.

**Figure 3:**
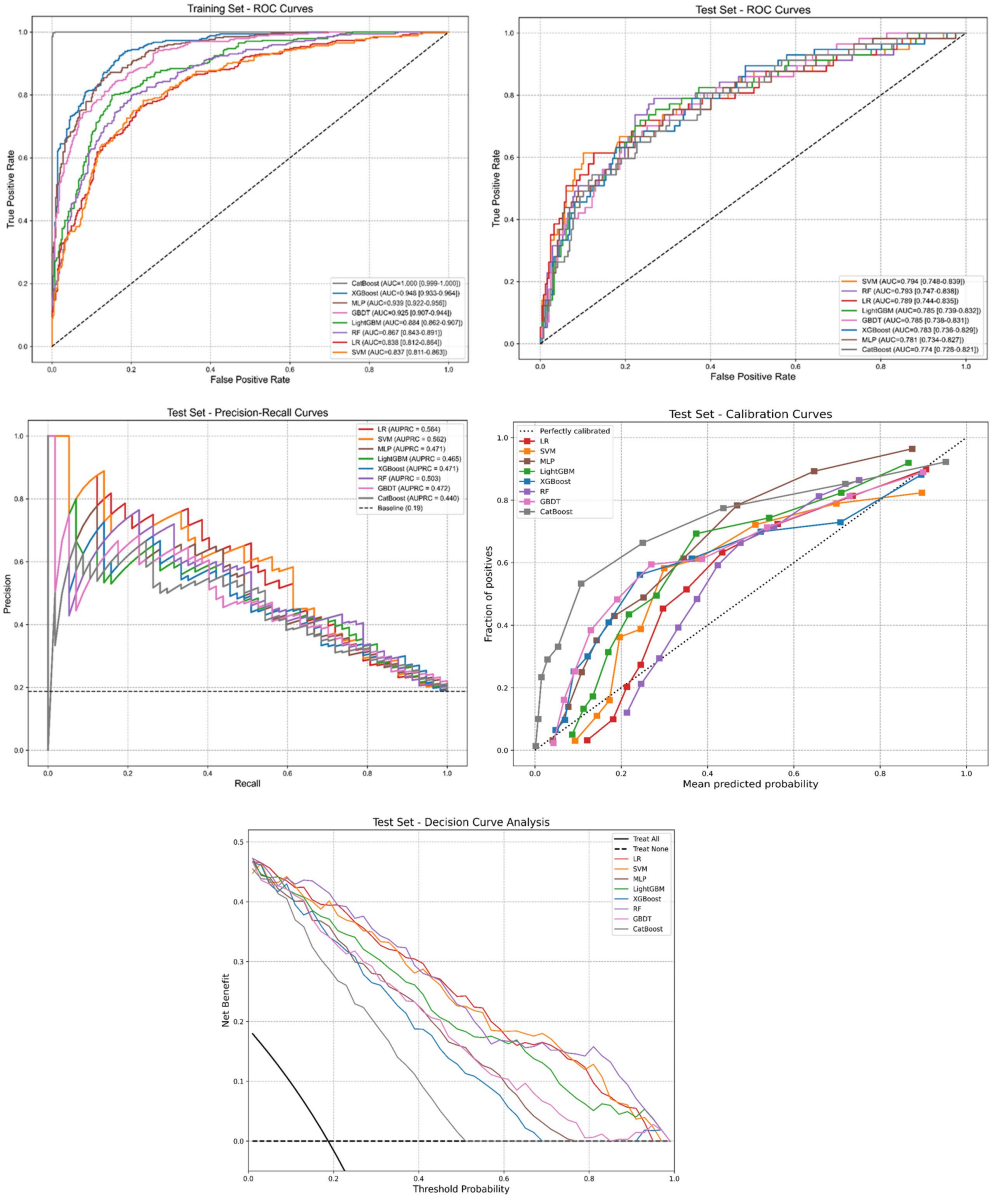
The performance and comparison of eight different predictive models. **(A)** ROC curves for the training set; **(B)** ROC curves for the test set; **(C)** precision-recall curves for the test set; **(D)** calibration curve for the test set; **(E)** decision curve analysis for the test set.

Eight machine learning models were employed to predict the occurrence of SAP among older patients of AIS. The performance of these models is illustrated in Figure 3, which shows the receiver operating characteristic curve (ROC), precision-recall (PR) curve, Brier score, calibration curve, and decision curve analysis (DCA). The performance of all eight models on the training and test sets is summarized in Table 2. Overall, the SVM model demonstrated the most consistent and robust predictive performance and was therefore selected as the final model due to its generalization stability, smallest training-test gap, and the intrinsic interpretability of its linear kernel, which shows by the SHAP analysis. The test set of the SVM model exhibited acceptable discriminative capability, with an AUC of 0.794 (95% CI 0.748–0.839), an accuracy of 0.773, a sensitivity of 0.667, a precision of 0.432, a specificity of 0.798, a Brier score of 0.158 (95% CI: 0.136–0.180), and an F1 score of 0.524.

**Table 2:** Detailed performance metrics of various machine learning models for predicting SAP risk in older patients with AIS across training and test sets.

Performance	Metrics	XGBoost	SVM	LightGBM	Logistic regression	Random forest	GBDT	MLP	CatBoost
Training	Accuracy	0.858	0.771	0.820	0.765	0.798	0.837	0.862	0.997
	Sensitivity	0.831	0.759	0.785	0.727	0.797	0.799	0.843	0.997
	Specificity	0.879	0.781	0.849	0.795	0.800	0.867	0.877	0.998
	Precision	0.703	0.575	0.649	0.558	0.596	0.677	0.739	0.995
	F1-score	0.839	0.747	0.795	0.733	0.778	0.814	0.844	0.997
	AUC (95%CI)	0.948 [0.933–0.964]	0.837 [0.811–0.863]	0.884 [0.862–0.907]	0.838 [0.812–0.864]	0.867 [0.812–0.864]	0.925 [0.907–0.944]	0.939 [0.922–0.956]	1.000 [0.999–1.000]
	Brier score (95%CI)	0.095 [0.091–0.112]	0.163 [0.149–0.177]	0.140 [0.127–0.152]	0.165 [0.153–0.177]	0.169 [0.160–0.178]	0.115 [0.103–0.126]	0.105 [0.131–0.158]	0.010 [0.008–0.011]
Test	Accuracy	0.779	0.773	0.785	0.780	0.763	0.785	0.781	0.774
	Sensitivity	0.579	0.667	0.632	0.684	0.711	0.632	0.614	0.579
	Specificity	0.844	0.798	0.829	0.802	0.789	0.829	0.846	0.824
	Precision	0.467	0.432	0.457	0.443	0.465	0.457	0.457	0.436
	F1-score	0.511	0.524	0.507	0.574	0.535	0.511	0.493	0.477
	AUC (95%CI)	0.783 [0.736–0.829]	0.794 [0.748–0.839]	0.785 [0.739–0.832]	0.789 [0.744–0.835]	0.793 [0.747–0.838]	0.785 [0.729–0.823]	0.781 [0.734–0.827]	0.774 [0.728–0.821]
	Brier score (95%CI)	0.160 [0.134–0.186]	0.158 [0.136–0.180]	0.162 [0.139–0.185]	0.172 [0.151–0.195]	0.183 [0.169–0.202]	0.163 [0.138–0.193]	0.150 [0.125–0.174]	0.158 [0.131–0.191]

Among all models, the SVM demonstrated a competitive AUC of 0.794 in the test set. Although other models, such as logistic regression and LightGBM, also performed well, the SVM was selected as the final predictive model based on its overall stability and minimal overfitting. Specifically, CatBoost achieved a training AUC of 1.000 but only 0.783 on the test set, indicative of overfitting. In contrast, linear models showed substantially smaller training-test AUC gaps: 0.044 for SVM and 0.049 for logistic regression, suggesting that simpler models are more appropriate for this dataset size and clinical prediction task. DeLong tests confirmed no statistically significant differences among the top-performing models (all *p* > 0.05), supporting a measured interpretation of SVM’s performance (Supplementary Table 2).

To further assess the model’s generalizability, we performed temporal validation by training the model on patients admitted from 2018 to 2021 (*n* = 707) and testing it on patients admitted from 2022 to 2023 (*n* = 304). The SVM model achieved an AUC of 0.789 (95% CI: 0.714–0.856) and a Brier score of 0.121 (95% CI: 0.096–0.150) in temporal validation, demonstrating consistent predictive performance over time.

### 3.3 Model explainability

**Figure 4:**
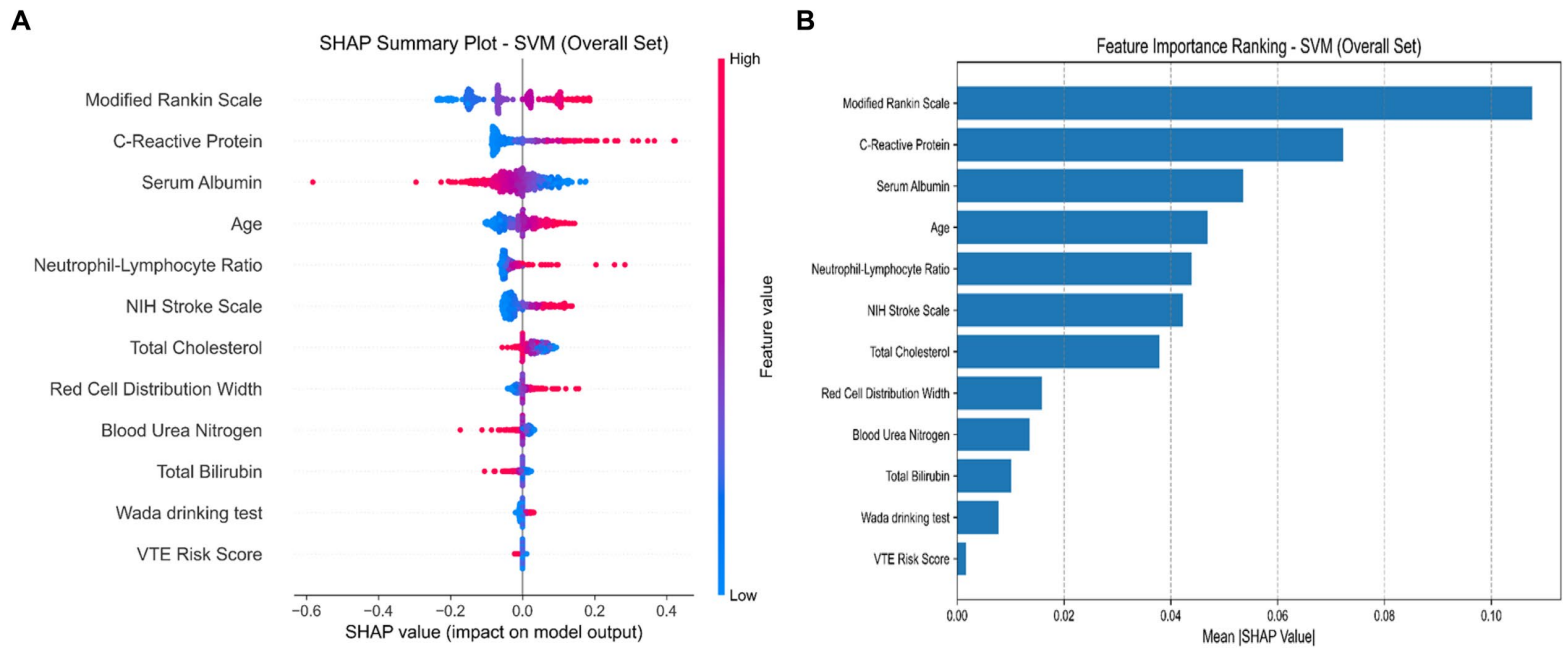
SHAP of the model. **(A)** Characteristic attributes in SHAP. The abscissa is the SHAP value, and each line denotes a feature. Higher eigenvalues are indicated by red dots, and lower eigenvalues are indicated by blue dots. **(B)** Importance ranking plot of features of the SVM model.

In these test data, we calculated the SHAP values for each feature variable to assess their contribution to the prediction results. This study assessed the relative significance of various factors influencing the susceptibility to SAP. Figure 4A visually represented the characteristic attributes in SHAP. The abscissa is the SHAP value, and each line denotes a feature. Higher eigenvalues are indicated by red dots, and lower eigenvalues are indicated by blue dots. Although linear SVM coefficients directly provide global feature importance, SHAP values were additionally computed to obtain instance-level contributions for individualized clinical explanation. The SHAP analysis provided invaluable insights into our predictive model for SAP, identifying the most influential factors in descending order of importance. The impact of the 12 features in the importance ranking on prediction outcomes is illustrated in Figure 4B. Specifically, mRS score, CRP, NLR, NIHSS score, advancing age, total bilirubin, Wada Drinking Test, BUN, VTE Risk Score, and RDW exhibit positive contributions to the predictive results, while serum albumin and total cholesterol demonstrate negative influences on the model’s output.

### 3.4 Clinical application

We implemented the application of the prediction model through a deployable web platform using Streamlit^1^ and uploaded the source code to GitHub. This provides an online tool for assessing the risk of SAP in older patients. By directly entering clinical feature data into the designated text fields on the webpage, users can easily obtain the desired prediction results.

## 4 Discussion

This single-center study developed and internally validated an interpretable SVM-based model for predicting SAP in older AIS patients using 12 routinely available clinical and laboratory features. The observed incidence of SAP varies widely (3.90–55.60%) owing to the heterogeneity of study populations, settings, and diagnostic criteria (26–29). Our study developed an interpretable SVM-based machine learning model for effectively predicting the risk of SAP in older patients with AIS using routinely available clinical and laboratory data. In this retrospective study, we found an 18.79% incidence of pneumonia in older patients with acute stroke. Using eight machine learning models, we evaluated the effectiveness of predicting SAP, and utilized SHAP analysis to identify key predictors. The result of the DeLong tests confirmed that no model significantly outperformed the others. Among these models, the SVM demonstrated the smallest performance gap between training and test sets and produced the most stable performance model in our comparison, with an AUC of 0.794 and acceptable precision, sensitivity, and F1-score values. SHAP analysis revealed that 12 key feature variables, including CRP, mRS score, NIHSS score, NLR, BUN, Wada drinking test score, VTE risk score, RDW, total bilirubin and age, were positively associated with an increased risk of SAP, whereas serum albumin and total cholesterol were negatively associated with the risk of SAP.

Although the SVM model demonstrated acceptable discrimination with an AUC of 0.794, the F1 score of 0.524 and precision of 0.432 on the test set, indicate that approximately 57% of patients predicted to develop SAP would not actually do so. This false-positive rate has significant clinical implications. A false-positive prediction could lead to unnecessary administration of prophylactic antibiotics, potentially contributing to antimicrobial resistance and other adverse drug events. Thus, we emphasize that this model should be positioned as a screening tool to identify older AIS patients who need to enhance clinical monitoring and a bundle of evidence-based, non-pharmacological preventive measures, including strict dysphagia screening, oral care protocols, and elevation of the head of the bed, instead of taking medical measures immediately, such as a trigger for immediate antibiotic initiation.

Our results showed that older patients diagnosed with SAP had significantly higher mRS scores than those who did not. Several studies from other groups support our results, indicating that the mRS score is the most significant predictor of SAP (30–32). A higher mRS score indicates that the patient requires a longer period of bed rest, has a poorer cough reflex, and suffers from more severe swallowing dysfunction. Beyond its predictive utility, the mechanistic basis for the association between higher mRS scores and SAP in old patients deserves attention. Severe stroke may predispose to SAP through three interrelated mechanisms. Firstly, Stroke-induced immunosuppression triggers a Th1/Th2 shift and lymphocytopenia. It also impairs the antimicrobial function of neutrophils and monocytes (33). Secondly, Dysphagia is a common consequence of severe stroke which increases aspiration risk. A meta-analysis shows that dysphagia confers a 9.60-fold increased risk of pneumonia (95% CI: 5.75–16.04) (34). Thirdly, presbyphagia is an age-related decline in swallowing safety and efficiency. It is often caused by pharyngeal hypesthesia (35, 36). Immunosuppression, dysphagia, and reduced age-related compensatory reserve together led to a high mRS score in older stroke patients. This convergence explains the score’s robust prediction of SAP. Our results also showed that older patients diagnosed with SAP had significantly higher NIHSS scores than those who did not. Previous studies had also established a strong correlation between NIHSS score and the risk of developing SAP (37, 38). This likely reflects the fact that more severe neurological deficits directly impair airway protection, swallowing, and respiratory function.

Advanced age is also a risk factor for SAP. Consistent with previous studies, older adult stroke patients experience a gradual decline in immune function and resistance, leading to reduced stress tolerance and increased susceptibility to SAP (39, 40). Additionally, Assefa et al. (41) demonstrated that stroke patients aged ≥75 years had a 4-fold higher risk of developing SAP compared to those aged 18–44 years.

Inflammation markers reflect the occurrence of SAP in older stroke patients. We found that levels of C-reactive protein, a prototypical acute-phase reactant, were significantly elevated in older patients who subsequently developed SAP (42). The onset of acute ischemic stroke triggers the activation of the autonomic nervous system and stress axis, which in turn suppresses the immune response (43). This suppression leads to higher white blood cell counts and increased CRP levels, indicating an amplified inflammatory response and weakened immune defense, thus increasing the likelihood of SAP development (44, 45). Furthermore, we confirmed that NLR is one of the factors influencing the development of SAP in older patients. Various studies have demonstrated that peripheral blood inflammatory markers are closely associated with SAP, providing a scientific and theoretical foundation for the diagnosis and prediction of this disease (46, 47). Dynamic monitoring of the NLR can be used for early prevention, ultimately reducing the incidence of SAP (48). Our findings also indicate that BUN is a risk factor for SAP, because it is a marker associated with systemic disease. High values of BUN lead to high susceptibility to infection (38). Additionally, patients with higher VTE risk scores often present with multiple conditions that also predispose to SAP, such as advanced age, immobility, and acute infection, which may explain the positive association between VTE risk score and SAP observed in our model (49). RDW is an indicator reflecting the heterogeneity of red blood cell sizes. Elevated RDW is associated with inflammatory response, poor nutritional status, chronic diseases, and stress, all of which may indirectly increase the risk of SAP (50). These findings are consistent with those of previous studies, and our study further demonstrated a significant ‌positive correlation between these predefined risk factors and the incidence of SAP in older patients.

In contrast, the results of this study indicated that serum albumin levels were negatively correlated with the risk of SAP in older patients (51). Total bilirubin was identified as a positive contributor to SAP risk in the LASSO model. This finding illustrates an important feature of multivariable modeling: a variable may serve as a meaningful predictor when accounting for confounding effects, even if its marginal distribution does not differ between outcome groups. Albumin can stimulate immune cells to enhance the immune level of the human body, and it also has antioxidant and anti-inflammatory effects (52, 53). The association between high albumin levels and better functional outcomes, as well as reduced mortality in acute ischemic stroke, suggests that albumin may have a neuroprotective effect (54). Total cholesterol was also a key protective factor for SAP. Beltowski et al. (55) emphasized the neuroprotective effect created by the higher cholesterol. Thus, an improved cholesterol level reduced post-stroke complications in patients with increased lipid profile levels (56, 57).

Although these factors are associated with SAP, integrating them into a robust predictive model for clinical use remains challenging. Several previous studies on predicting SAP in stroke patients have explored various prediction models, such as logistic regression, SVM, and LightGBM (43, 58, 59). Since the older patients are at a higher risk of stroke and SAP, several recent studies have attempted to explore the specific risk factors for SAP in older patients. Wu et al. (60) demonstrated a strong correlation between SAP and Barthel Index scores. Another retrospective study by Cao et al. constructed an LR-based prediction model for SAP in older adult patients with hemorrhagic stroke. Age, smoking, low Glasgow Coma Scale (GCS) and Braden scores, and nasogastric tube feeding were identified as important predictive factors for SAP in these patients (61). However, these studies present several limitations, including relatively small sample sizes with limited representativeness and a narrow set of research variables, further restricting the practical applicability of the results. Thus, the early identification of SAP in older patients with AIS remains largely unexplored.

In this study, we developed an ML model employing eight models for SAP prediction. Our results indicate that SVM demonstrated competitive and stable performance compared with the other seven machine learning models in terms of accuracy, sensitivity, and F1-scores in the test set. The accuracy and AUROC values also performed well, as did the SVM in the test suite. A recent meta-analysis of machine learning models for SAP reported a pooled AUC of approximately 0.82 (6). Our model’s AUC of 0.794 is slightly lower but within the reported range; importantly, it was developed specifically for older patients, a subgroup where prediction may be inherently more challenging. The calibration curve revealed that the SVM closely followed the ideal line across the entire probability range, achieving a Brier score of 0.163 and 0.158 on the training and test sets, respectively, confirming acceptable calibration and predictive accuracy. The complete results for all models are provided in Supplementary Table 5. Decision curve analysis demonstrated that the SVM model provided a comparable net benefit across a clinically relevant range of threshold probabilities among eight models. SVM showed stable performance between training and test sets, leading us to choose it as the final predictive tool.

Compared with other risk assessment models, our machine-learning model offers several critical advantages while acknowledging certain disadvantages. The current study utilized a five-year cohort and a total of 1,011 patients, which provided a relatively large sample size for model development. Another key advantage is that this modeling framework, through LASSO selection, efficiently identifies key predictors and constructs a linear decision boundary. Our model integrates both laboratory results and indicators routinely examined at admission of the patient, which may help doctors identify the targeted population and adopt timely interventions to reduce the occurrence of poor outcomes more conveniently and intelligently. By integrating routine admission indicators with laboratory results, it provides a more individualized risk profile assessment, helping clinicians to identify high-risk patients and intervene earlier. To enhance clinical applicability, we developed a simple online platform that enables healthcare professionals to utilize machine learning models anytime and anywhere to identify the risk of SAP among older stroke patients, facilitating early detection and timely intervention, so that clinicians can easily estimate SAP risk in older stroke patients anytime and anywhere. While no tool can entirely replace clinical judgment, this system bridges the gap between advanced analytics and bedside decision-making, supporting personalized prevention strategies. In addition, decision curve analysis (DCA) further confirmed the model’s clinical utility, demonstrating a net benefit across a wide range of risk thresholds. Finally, this study specifically targets older patients with AIS aged ≥65 years, who have a high incidence of SAP and encounter substantial challenges in treatment decision-making owing to their increased risk of adverse outcomes.

Despite these advantages, several limitations must be recognized. First, although the sample size was relatively large, this study utilized data from a single hospital, and no external validation was conducted. Second, prevalent indicators of SAP identified in other studies, such as blood biomarkers associated with the systemic inflammatory response and immune changes, were not available in this study. Third, care process variables such as dysphagia screening protocols, head-of-bed elevation, oral care, and early mobilization were also not captured in our retrospective dataset. These factors are known determinants of SAP and their omission may affect model performance in settings with different care standards (62). Fourth, an in-depth analysis of SAP was not performed. Subgroup analyses of pneumonia severity, radiological features, and pathogenic agents were absent. Future studies should investigate whether incorporating novel predictors can enhance the predictive accuracy of the model. Subsequent efforts should focus on validating the model using the most current multicenter data. It is worth noting, however, that an AUC of 0.794 reflects moderate discriminatory ability. Though this level of performance may be acceptable in a clinical context, further improvements could be achieved by incorporating larger and more diverse datasets.

## 5 Conclusion

In this study, we demonstrate that an effective machine learning model can be constructed to predict the risk of SAP in older patients with AIS by combining data routinely available from clinical and laboratory settings. The results indicated that among the eight machine learning algorithms, the SVM model revealed the best and most stable predictive performance. SHAP analysis further improved the clinical interpretability of the model by elucidating the feature contributions. The mRS score, NIHSS score, CRP level, NLR, BUN level, Wada drinking test score, VTE risk score, RDW, total bilirubin, and age were identified as predictive factors for SAP in older patients with AIS, whereas serum albumin and total cholesterol decreased the risk of SAP. This interpretable SVM-based machine learning model and online prediction platform can facilitate early identification of SAP risk in older patients with AIS. Early identification of high-risk patients can help clinicians implement targeted prophylactic measures, which may reduce the incidence of SAP and improve patient outcomes.

## Data Availability

The raw data supporting the conclusions of this article will be made available by the authors, without undue reservation.

## Footnotes

1
https://63cokcyxjt9gjngmxpwrfn.streamlit.app
