# Supplementary figures and images for: Development and validation of a machine learning model for predicting stroke-associated pneumonia in older patients with acute ischemic stroke
Source: Front Neurol. 2026 Jun 10;17:1801193. doi: 10.3389/fneur.2026.1801193 (PMC13290704; doi:10.3389/fneur.2026.1801193)

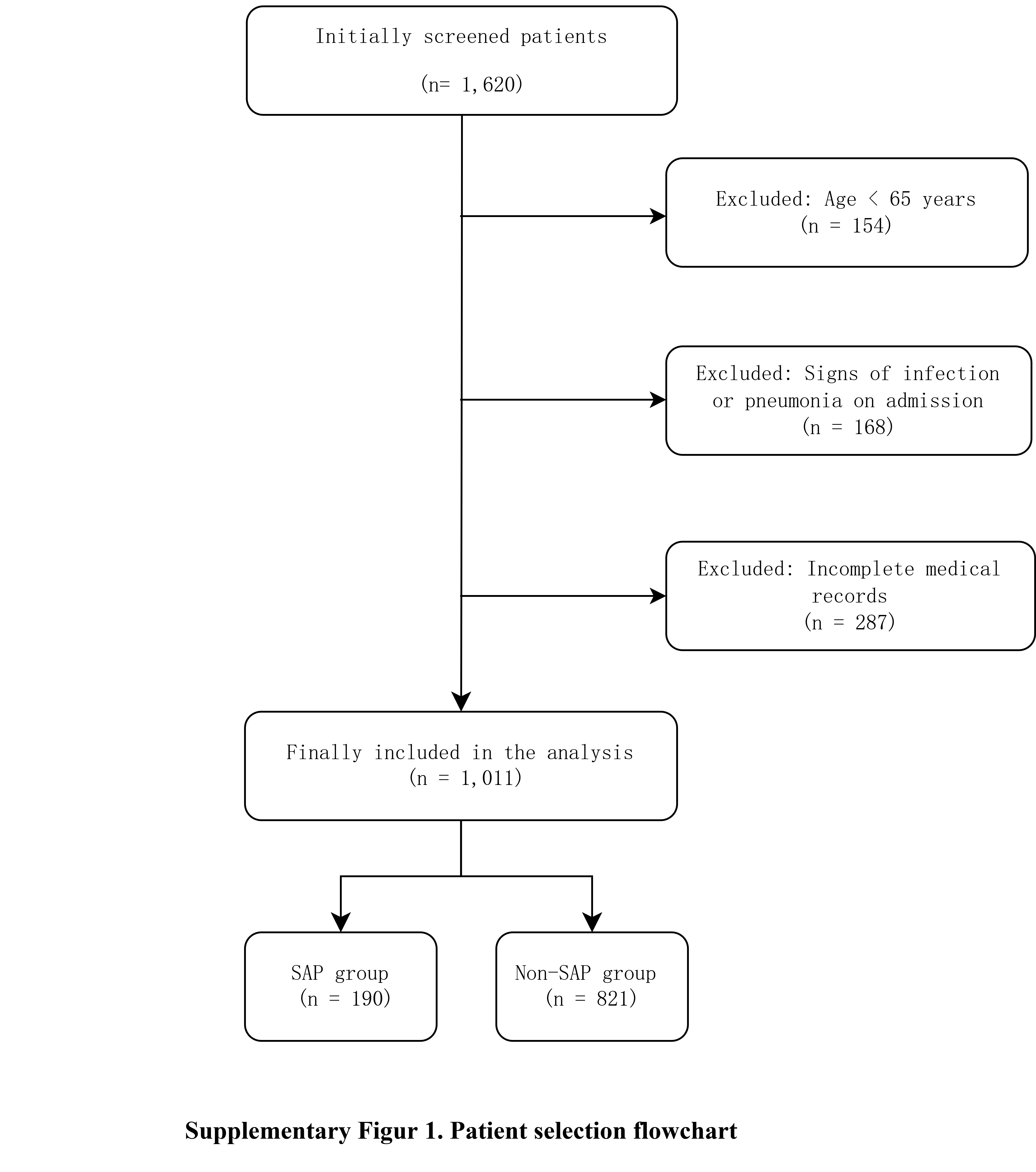

Supplement: Supplementary file 5 [file Image_1.png]

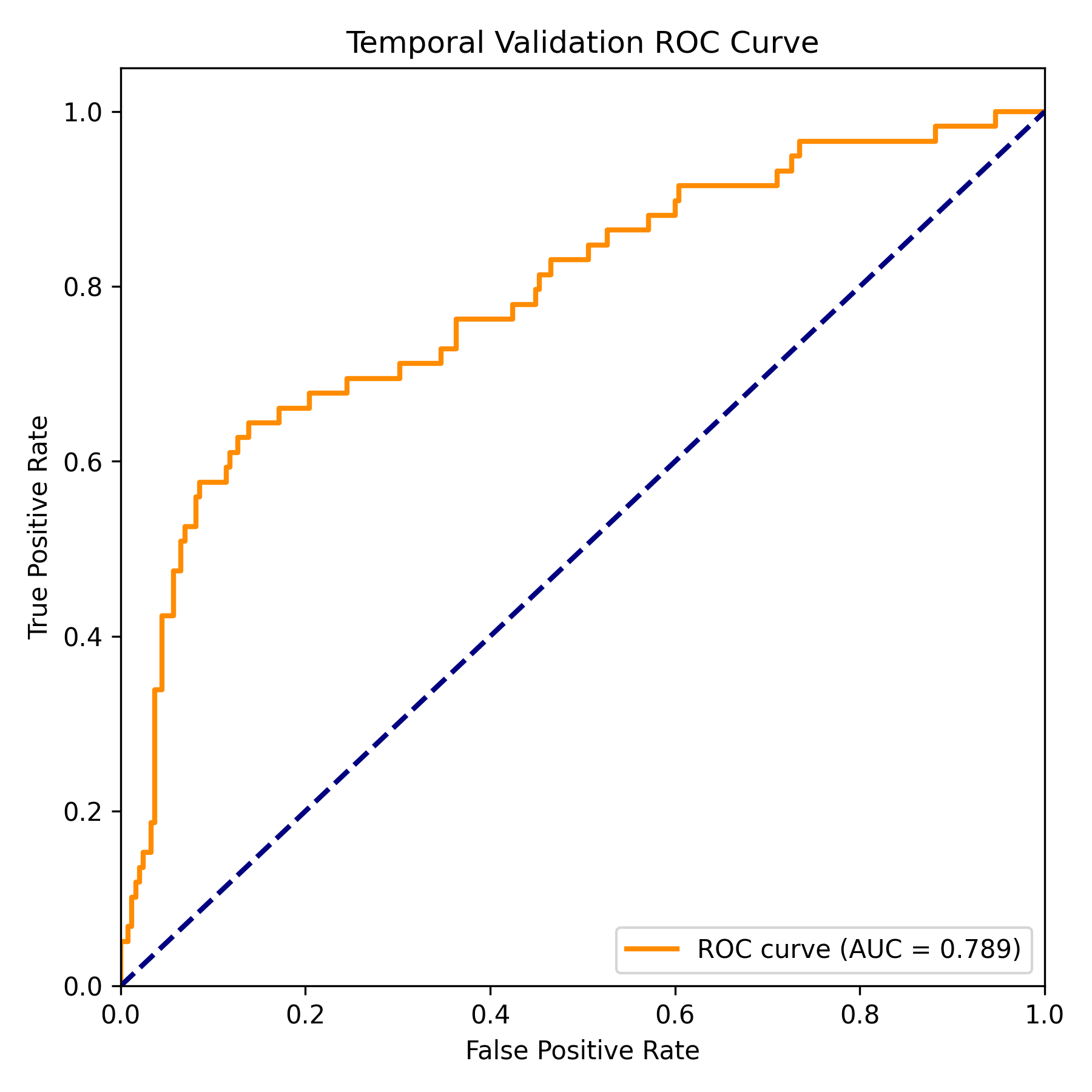

Supplement: Supplementary file 6 [file Image_2.png]
