# Supplementary material for: Development and validation of a machine learning model for predicting stroke-associated pneumonia in older patients with acute ischemic stroke
Source: Front Neurol. 2026 Jun 10;17:1801193. doi: 10.3389/fneur.2026.1801193 (PMC13290704; doi:10.3389/fneur.2026.1801193)
Supplement: Supplementary file 7 [file Supplementary_file_1.docx]

Input: dataset D with features X and binary outcome y

Output: best_model, performance metrics on test set

1. Load D and set random seed (42)

2. Split D into training set (70%) and test set (30%) using stratified sampling

X_train, X_test, y_train, y_test ← train_test_split(X, y, stratify=y)

3. Feature selection (only on training data):

- Normalize X_train with StandardScaler (fit only on X_train)

- Perform 10-fold LassoCV to select 10–12 top features with non-zero coefficients

- selected_features ← columns of X_train with largest |coefficients|

- X_train ← X_train[selected_features]

- X_test ← X_test[selected_features]

4. Define eight classifiers (LR, SVM, MLP, LightGBM, XGBoost, RF, GBDT, CatBoost)

For each classifier, define hyperparameter grid (see Supplementary Table 1)

5. For each classifier:

a. Build an ImbPipeline:

[StandardScaler(), SMOTE(random_state=42), classifier]

b. Wrap the pipeline in GridSearchCV with 5-fold StratifiedKFold

(cv = StratifiedKFold(5, shuffle=True, random_state=42),

scoring = 'roc_auc')

c. Fit GridSearchCV on X_train, y_train

IMPORTANT: Inside each fold, the pipeline:

i. Fits StandardScaler on the training fold only → transforms training & validation fold

ii. Applies SMOTE only on the training fold (after scaling)

iii. Trains classifier on the balanced, scaled training fold

iv. Predicts probabilities on the untouched validation fold

→ This prevents any information leakage from validation/test data into training.

d. Select best estimator from GridSearchCV

e. Predict on X_test using the final pipeline:

y_test_prob ← best_pipe.predict_proba(X_test)[:,1]

y_test_pred ← best_pipe.predict(X_test)

f. Calculate metrics on y_test vs. y_test_pred / y_test_prob:

- AUC with 95% bootstrap CI (2000 resamples)

- Brier score with 95% bootstrap CI

- Accuracy, Sensitivity, Specificity, Precision, F1

- Optimal threshold (maximising F1 on training set)

- Hosmer–Lemeshow χ² and p-value

- Calibration slope and intercept

6. Compare all models using Bootstrap DeLong test for paired AUC differences

7. Select best model as the one with highest test-set Optimized F1

8. Generate SHAP values on test set using appropriate explainer

(TreeExplainer for trees, KernelExplainer for SVM, etc.)

Output SHAP summary plots and CSV tables of SHAP values

9. Save best model, selected features, and performance matrices to disk
