# Supplementary material for: Development and validation of a machine learning model for predicting stroke-associated pneumonia in older patients with acute ischemic stroke
Source: Front Neurol. 2026 Jun 10;17:1801193. doi: 10.3389/fneur.2026.1801193 (PMC13290704; doi:10.3389/fneur.2026.1801193)
Supplement: Supplementary file 8 [file Supplementary_file_2.docx]

| **Section/Topic Item Development Checklist item**  **/ evaluation**^1^ | | | | **Reported on page** |
| --- | --- | --- | --- | --- |
| **TITLE** | | | |  |
| *Title* | 1 | D;E | Identify the study as developing or evaluating the performance of a multivariable prediction model, the target population, and the outcome to be predicted | Development and Validation of a Machine Learning Model for Predicting Stroke-Associated Pneumonia in Older Patients with Acute Ischemic Stroke |
| **ABSTRACT** | | | | |
| *Abstract* | 2 | D;E | See TRIPOD+AI for Abstracts checklist | Abstract section; includes background, objective, methods, results (AUC 0.795, Brier 0.156, 12 features), and conclusion |
| **INTRODUCTION** | | | | |
| *Background* | 3a | D;E | Explain the healthcare context (including whether diagnostic or prognostic) and rationale for developing or evaluating the prediction model, including references to existing models | Introduction, paragraphs 1-3; describes SAP as a common complication; references existing models including AIS-APS, PANTHERIS, A2DS2 scores |
|  | 3b | D;E | Describe the target population and the intended purpose of the prediction model in the context of the care pathway, including its intended users (e.g., healthcare professionals, patients, public) | Introduction, paragraph 4; target population: older patients (≥65 years) with AIS; intended for clinical decision support |
|  | 3c | D;E | Describe any known health inequalities between sociodemographic groups | Not explicitly discussed in manuscript |
| *Objectives* | 4 | D;E | Specify the study objectives, including whether the study describes the development or validation of a prediction model (or both) | Introduction, last paragraph: "This study aimed to develop and validate an explainable machine-learning model for identifying the risk of SAP in older patients with AIS using routinely available clinical features" |
| **METHODS** | | | | |
| *Data* | 5a | D;E | Describe the sources of data separately for the development and evaluation datasets (e.g., randomised trial, cohort, routine care or registry data), the rationale for using these data, and representativeness of the data | Single-center retrospective data from Zhejiang Hospital, Hangzhou, China; consecutive admissions |
|  | 5b | D;E | Specify the dates of the collected participant data, including start and end of participant accrual; and, if applicable, end of follow-up | September 1, 2018 to December 31, 2023 |
| *Participants* | 6a | D;E | Specify key elements of the study setting (e.g., primary care, secondary care, general population)  including the number and location of centres | certified stroke unit that provides various medical services, including acute stroke care, intravenous thrombolysis, endovascular therapy, intensive monitoring, and early rehabilitation |
|  | 6b | D;E | Describe the eligibility criteria for study participants | aged ≥65 years; AIS confirmed by CT or MRI within 24 hours of admission; CDC-based SAP diagnostic criteria; exclusion criteria listed |
|  | 6c | D;E | Give details of any treatments received, and how they were handled during model development or evaluation, if relevant | not specifically handled as predictors |
| *Data preparation* | 7 | D;E | Describe any data pre-processing and quality checking, including whether this was similar across  relevant sociodemographic groups | Z-score normalization; SMOTE applied within cross-validation folds; patients with missing values excluded |
| *Outcome* | 8a | D;E | Clearly define the outcome that is being predicted and the time horizon, including how and when assessed, the rationale for choosing this outcome, and whether the method of outcome assessment is  consistent across sociodemographic groups | SAP defined per CDC criteria as lower respiratory tract infection within 7 days of stroke onset |
|  | 8b | D;E | If outcome assessment requires subjective interpretation, describe the qualifications and demographic characteristics of the outcome assessors | CDC criteria are objective; assessment performed by certified physicians |
|  | 8c | D;E | Report any actions to blind assessment of the outcome to be predicted | Not explicitly stated |
| *Predictors* | 9a | D | Describe the choice of initial predictors (e.g., literature, previous models, all available predictors) and  any pre-selection of predictors before model building | 32 variables selected after surveying previous SAP risk factor studies and discussing with physicians |
|  | 9b | D;E | Clearly define all predictors, including how and when they were measured (and any actions to blind assessment of predictors for the outcome and other predictors) | detailed list including demographics, comorbidities, laboratory tests, and admission assessments (NIHSS, Wada, VTE, mRS) |
|  | 9c | D;E | If predictor measurement requires subjective interpretation, describe the qualifications and demographic characteristics of the predictor assessors | NIHSS and mRS scored by certified doctors through face-to-face interviews |
| *Sample size* | 10 | D;E | Explain how the study size was arrived at (separately for development and evaluation), and justify that  the study size was sufficient to answer the research question. Include details of any sample size calculation | 1,011 eligible patients from 1,620 stroke patients after exclusion criteria; no formal sample size calculation reported |
| *Missing data* | 11 | D;E | Describe how missing data were handled. Provide reasons for omitting any data | Patients with any missing values for any of the 32 initial variables were excluded |
| *Analytical methods* | 12a | D | Describe how the data were used (e.g., for development and evaluation of model performance) in the analysis, including whether the data were partitioned, considering any sample size requirements | 7:3 random split (training/testing); 5-fold cross-validation for training; temporal validation (2018-2021 train, 2022-2023 test) |
|  | 12b | D | Depending on the type of model, describe how predictors were handled in the analyses (functional form,  rescaling, transformation, or any standardisation). | Z-score normalization; SMOTE; LASSO for feature selection (12 of 32 variables selected) |
|  | 12c | D | Specify the type of model, rationale^2^, all model-building steps, including any hyperparameter tuning,  and method for internal validation | 8 models (LR, SVM, LightGBM, XGBoost, CatBoost, GBDT, MLP, RF); Grid Search for hyperparameter optimization; 5-fold CV |
|  | 12d | D;E | Describe if and how any heterogeneity in estimates of model parameter values and model performance was handled and quantified across clusters (e.g., hospitals, countries). See TRIPOD-Cluster for  additional considerations^3^ | Single-center study; not applicable |
|  | 12e | D;E | Specify all measures and plots used (and their rationale) to evaluate model performance (e.g., discrimination, calibration, clinical utility) and, if relevant, to compare multiple models | confusion matrix, F1-score, Brier score, AUC, calibration curves, DCA, DeLong test |
|  | 12f | E | Describe any model updating (e.g., recalibration) arising from the model evaluation, either overall or for particular sociodemographic groups or settings | Not performed; internal validation only |
|  | 12g | E | For model evaluation, describe how the model predictions were calculated (e.g., formula, code, object, application programming interface) | Streamlit web platform provided; code on GitHub |
| *Class imbalance* | 13 | D;E | If class imbalance methods were used, state why and how this was done, and any subsequent methods to  recalibrate the model or the model predictions | SMOTE applied due to SAP incidence of 18.79%; applied within cross-validation folds only |
| *Fairness* | 14 | D;E | Describe any approaches that were used to address model fairness and their rationale | Not explicitly discussed |
| *Model output* | 15 | D | Specify the output of the prediction model (e.g., probabilities, classification). Provide details and  rationale for any classification and how the thresholds were identified | Default threshold of 0.5 used for binary classification; sensitivity analysis with Youden index in Supplementary Table 4 |

^1^ D=items relevant only to the development of a prediction model; E=items relating solely to the evaluation of a prediction model; D;E=items applicable to both the development and evaluation of a prediction model

^2^ Separately for all model building approaches.

^3^ TRIPOD-Cluster is a checklist of reporting recommendations for studies developing or validating models that explicitly account for clustering or explore heterogeneity in model performance (eg, at different hospitals or centres). Debray et al, BMJ 2023; 380: e071018 [DOI: 10.1136/bmj-2022-071018]

| *Training versus*  *evaluation* | 16 | D;E | Identify any differences between the development and evaluation data in healthcare setting, eligibility  criteria, outcome, and predictors | Same source (single center); temporal validation uses different time periods; Table 1 shows no significant demographic differences between training and test sets |
| --- | --- | --- | --- | --- |
| *Ethical approval* | 17 | D;E | Name the institutional research board or ethics committee that approved the study and describe the participant-informed consent or the ethics committee waiver of informed consent | Ethics committee of Zhejiang Hospital (document number: 2024linshen111K); informed consent waived due to retrospective nature |
| **OPEN SCIENCE** | | | | |
| *Funding* | 18a | D;E | Give the source of funding and the role of the funders for the present study | Not explicitly stated in the manuscript |
| *Conflicts of interest* | 18b | D;E | Declare any conflicts of interest and financial disclosures for all authors | The authors declare that the research was conducted in the absence of any commercial or financial relationships |
| *Protocol* | 18c | D;E | Indicate where the study protocol can be accessed or state that a protocol was not prepared | Not stated |
| *Registration* | 18d | D;E | Provide registration information for the study, including register name and registration number, or state  that the study was not registered | Not stated |
| *Data sharing* | 18e | D;E | Provide details of the availability of the study data | Not explicitly stated beyond GitHub code availability |
| *Code sharing* | 18f | D;E | Provide details of the availability of the analytical code^4^ | GitHub repository URL provided; analysis code, preprocessing pipelines, and trained model weights publicly available |
| **PATIENT & PUBLIC INVOLVEMENT** | | | | |
| *Patient & Public Involvement* | 19 | D;E | Provide details of any patient and public involvement during the design, conduct, reporting, interpretation, or dissemination of the study or state no involvement. | Not stated |
| **RESULTS** | | | | |
| *Participants* | 20a | D;E | Describe the flow of participants through the study, including the number of participants with and without the outcome and, if applicable, a summary of the follow-up time. A diagram may be helpful. | Section 3.1 and Supplementary Figure 1: 1,620 stroke patients and 1,011 included; 190 (18.79%) with SAP |
|  | 20b | D;E | Report the characteristics overall and, where applicable, for each data source or setting, including the key dates, key predictors (including demographics), treatments received, sample size, number of outcome events, follow-up time, and amount of missing data. A table may be helpful. Report any  differences across key demographic groups. | median age 76 (IQR 70-83), 67.6% male; Table 1 provides detailed characteristics with comparisons between SAP and non-SAP groups |
|  | 20c | E | For model evaluation, show a comparison with the development data of the distribution of important predictors (demographics, predictors, and outcome). | Table 1 reports training vs test set comparisons; differences noted in comorbidities, laboratory tests, and admission assessments |
| *Model development* | 21 | D;E | Specify the number of participants and outcome events in each analysis (e.g., for model development, hyperparameter tuning, model evaluation) | Training set (70%, n≈708), test set (30%, n≈303); temporal validation: 2018-2021 (n=707) train, 2022-2023 (n=304) test |
| *Model specification* | 22 | D | Provide details of the full prediction model (e.g., formula, code, object, application programming interface) to allow predictions in new individuals and to enable third-party evaluation and implementation, including any restrictions to access or re-use (e.g., freely available, proprietary)^5^ | GitHub repository provided; SVM with linear kernel (C=0.05); 12 LASSO-selected features; Streamlit app for implementation |
| *Model performance* | 23a | D;E | Report model performance estimates with confidence intervals, including for any key subgroups (e.g., sociodemographic). Consider plots to aid presentation. | SVM test set: AUC 0.794 (95% CI 0.748-0.863), accuracy 0.773, sensitivity 0.667, specificity 0.798, F1 0.524, Brier 0.156 |
|  | 23b | D;E | If examined, report results of any heterogeneity in model performance across clusters. See TRIPOD  Cluster for additional details^3^. | Single-center study; not examined |
| *Model updating* | 24 | E | Report the results from any model updating, including the updated model and subsequent performance | Not performed |
| **DISCUSSION** | | | | |
| *Interpretation* | 25 | D;E | Give an overall interpretation of the main results, including issues of fairness in the context of the  objectives and previous studies | Discussion, paragraph 1: SVM-based model can effectively predict SAP; SHAP analysis identified key predictors |
| *Limitations* | 26 | D;E | Discuss any limitations of the study (such as a non-representative sample, sample size, overfitting, missing data) and their effects on any biases, statistical uncertainty, and generalizability | Discussion, limitations paragraph: single-center design, no external validation, missing care process variables, no subgroup analyses of pneumonia severity, moderate AUC of 0.794 |
| *Usability of the model in the context of current care* | 27a | D | Describe how poor quality or unavailable input data (e.g., predictor values) should be assessed and handled when implementing the prediction model | N/A |
|  | 27b | D | Specify whether users will be required to interact in the handling of the input data or use of the model,  and what level of expertise is required of users | web platform developed; users enter clinical feature data into text fields for prediction |
|  | 27c | D;E | Discuss any next steps for future research, with a specific view to applicability and generalizability of  the model | Discussion, limitations paragraph: need for multicenter external validation, incorporating novel predictors |

From: Collins GS, Moons KGM, Dhiman P, et al. *BMJ* 2024;385:e078378. doi:10.1136/bmj-2023-078378

^4^ This relates to the analysis code, for example, any data cleaning, feature engineering, model building, evaluation.

^5^ This relates to the code to implement the model to get estimates of risk for a new individual.
