# Supplementary material for: Development and validation of a machine learning model for predicting stroke-associated pneumonia in older patients with acute ischemic stroke
Source: Front Neurol. 2026 Jun 10;17:1801193. doi: 10.3389/fneur.2026.1801193 (PMC13290704; doi:10.3389/fneur.2026.1801193)
Supplement: Supplementary file 9 [file Table_1.docx]

Supplementary Table 1-1: Complete hyperparameter grid and fixed settings for all models

| **Model** | **Hyperparameter** | **Search / Fixed values** | **Notes** |
| --- | --- | --- | --- |
| **LR** (Logistic Regression) | C (inverse regularization) | [0.001, 0.01, 0.1, 1, 10] | Searched |
|  | Penalty | [‘l1’, ‘l2’] | Searched |
|  | Solver | ‘liblinear’ | Fixed (required for L1) |
|  | Class weight | ‘balanced’ | Fixed |
|  | Maximum iterations | 1000 | Fixed |
|  | Random state | 42 | Fixed |
| **SVM** (Linear SVC) | C (inverse regularization) | [0.001, 0.01, 0.1, 1, 10] | Searched |
|  | Gamma | [‘scale’, ‘auto’] | Searched |
|  | Kernel | ‘linear’ | Fixed |
|  | Probability | True | Fixed (to enable predict_proba) |
|  | Class weight | ‘balanced’ | Fixed |
|  | Random state | 42 | Fixed |
| **MLP** (Multi‑layer Perceptron) | Hidden layer sizes | [(30,), (50,), (30,15), (50,25)] | Searched |
|  | Alpha (L2 penalty) | [0.001, 0.01, 0.1] | Searched |
|  | Learning rate (initial) | [0.001, 0.01] | Searched |
|  | Early stopping | True | Fixed |
|  | Maximum iterations | 1000 | Fixed |
|  | Random state | 42 | Fixed |
| **LightGBM** | Learning rate | [0.01, 0.05] | Searched |
|  | Number of estimators | [200, 300] | Searched |
|  | Number of leaves | [7, 15, 31] | Searched |
|  | Maximum depth | [3, 5, 7] | Searched (default = -1, no limit) |
|  | Minimum child samples | [50, 100] | Searched |
|  | L1 regularization (reg_alpha) | [1.0, 3.0, 5.0] | Searched |
|  | L2 regularization (reg_lambda) | [1.0, 3.0, 5.0] | Searched |
|  | Subsample | [0.6, 0.8] | Searched |
|  | Verbosity | -1 | Fixed (silent) |
|  | Random state | 42 | Fixed |
| **XGBoost** | Learning rate | [0.01, 0.05] | Searched |
|  | Maximum depth | [3, 5, 7] | Searched |
|  | Number of estimators | [100, 200] | Searched |
|  | Subsample | [0.6, 0.8] | Searched |
|  | Column subsample (by tree) | [0.6, 0.8] | Searched |
|  | L1 regularization (alpha) | [1.0, 3.0] | Searched |
|  | L2 regularization (lambda) | [1.0, 3.0] | Searched |
|  | Gamma (min split loss) | [0.1, 0.5] | Searched |
|  | Evaluation metric | ‘logloss’ | Fixed |
|  | Use label encoder | False | Fixed |
|  | Random state | 42 | Fixed |
| **RF** (Random Forest) | Number of estimators | [100, 200, 300] | Searched |
|  | Maximum depth | [5, 10, 15] | Searched |
|  | Minimum samples split | [10, 20] | Searched |
|  | Minimum samples leaf | [4, 8] | Searched |
|  | Maximum features | [‘sqrt’, ‘log2’] | Searched |
|  | Class weight | ‘balanced’ | Fixed |
|  | Bootstrap | True (default) | Fixed |
|  | Random state | 42 | Fixed |
| **GBDT** (Gradient Boosting) | Learning rate | [0.01, 0.05] | Searched |
|  | Number of estimators | [100, 200] | Searched |
|  | Maximum depth | [3, 5] | Searched |
|  | Minimum samples leaf | [20, 50] | Searched |
|  | Subsample | [0.6, 0.8] | Searched |
|  | Loss function | ‘log_loss’ (default) | Fixed |
|  | Random state | 42 | Fixed |
| **CatBoost** | Iterations | [200, 500] | Searched |
|  | Learning rate | [0.01, 0.05, 0.1] | Searched |
|  | Depth | [4, 6, 8] | Searched |
|  | L2 leaf regularization | [3.0, 5.0, 7.0] | Searched |
|  | Border count | 64 | Fixed (single value) |
|  | Subsample | [0.6, 0.8] | Searched |
|  | Verbosity | False | Fixed (silent) |
|  | Random seed | 42 | Fixed |
| **Pipeline‑level settings** (applied to all models) | StandardScaler | with mean centering & unit variance | Fixed |
|  | SMOTE (synthetic minority over‑sampling) | k_neighbors = min(5, n_minority−2) | Fixed (applied only to training folds during CV) |
|  | Cross‑validation | 5‑fold stratified, random_state=42 | Fixed |
|  | Scoring metric for grid search | ROC AUC | Fixed |

Supplementary Table 1-2 Hyperparameters of the models

| model | | hyperparameters |
| --- | --- | --- |
| **LR** | C=0.001, penalty=l2, solver=liblinear | |
| **SVM** | C=0.001, gamma=scale, kernel=linear | |
| **MLP** | alpha=0.1, hidden_layer_sizes=(50,), learning_rate_init=0.001 | |
| **LightGBM** | learning_rate=0.01, max_depth=7, n_estimators=300, num_leaves=15 | |
| **XGBoost** | learning_rate=0.01, max_depth=7, n_estimators=200, gamma=0.5 | |
| **RF** | max_depth=5, n_estimators=200, max_features=sqrt | |
| **GBDT** | learning_rate=0.01, max_depth=5, n_estimators=200, subsample=0.6 | |
| **CatBoost** | depth=8, iterations=200, learning_rate=0.05, l2_leaf_reg=7.0 | |
